# Negative Mood Increases Selective Attention to Negatively Valenced Body Parts in Female Adolescents with Anorexia Nervosa

**DOI:** 10.1371/journal.pone.0154462

**Published:** 2016-04-28

**Authors:** Jennifer Svaldi, Caroline Bender, Detlef Caffier, Viliana Ivanova, Nina Mies, Christian Fleischhaker, Brunna Tuschen-Caffier

**Affiliations:** 1 Department of Psychology, University of Tübingen, Tübingen, Germany; 2 Department of Psychology, University of Freiburg, Freiburg, Germany; 3 Department of Child and Adolescent Psychiatry and Psychotherapy, University Medical Centre Freiburg, Freiburg, Germany; Sichuan University, CHINA

## Abstract

**Objective:**

Previous research has yielded evidence of increased attentional processing of negatively valenced body parts in women with anorexia nervosa (AN), especially for those with high depressive symptomatology. The present study extended previous research by implementing an experimental mood manipulation.

**Method:**

In a within-subjects design, female adolescents with AN (*n* = 12) and an age matched female control group (CG; *n* = 12) were given a negative and a positive mood induction at a one-week interval. After each mood induction, participants underwent a 3-min mirror exposure, while their eye movements were recorded.

**Results:**

After the positive mood induction, both AN and CG participants displayed longer and more frequent gazes towards their self-defined most ugly relative to their self-defined most beautiful body part. However, after the negative mood induction, only females with AN were characterized by increased attention to their most ugly compared to their most beautiful body part, while CG participants’ attention distribution was balanced. Furthermore, in the negative (but not in the positive) mood induction condition gaze frequency and duration towards the most ugly body part was significantly stronger in the AN group relative to the CG.

**Discussion:**

The results emphasize the role of negative mood in the maintenance of pathological information processing of the self-body. This increased body-related negativity-bias during negative mood may lead to the persistence and aggravation of AN patients’ body image disturbance.

## Introduction

Anorexia nervosa (AN) is characterized by altered eating behavior and cognitive-affective disturbances regarding the body and the self. A core feature of AN is the restriction of energy intake relative to daily requirements, leading to excessive weight loss. Further key criteria are body image disturbances such as an intense fear of weight gain, disturbances in the way one’s body weight or shape is experienced, undue influence of weight and shape on self-evaluation and denial of the seriousness of the current low body weight (American Psychiatric Association, APA [1]).

Empirical evidence suggests that body image disturbances are a pivotal etiological [2–5] and maintenance [6–8] factor of eating disorders. According to schema-theoretical models [9, 10], body dissatisfaction in eating disorders results from maladaptive schemata regarding the self and the body. These maladaptive schemata are thought to influence the perception, attention, memory and interpretation of body-related information, thereby maintaining body image disturbances by guiding the scope of information being processed to schema-consistent contents. Notably, attentional biases are of particular importance, as they can automatically trigger a sequence of processes in the information processing stream, including body-related interpretation and evaluation.

Using the eye tracking methodology, a number of studies have yielded empirical evidence of these theoretical assumptions, even though the results have not always been consistent. In one study [11] adolescent females with AN differed from healthy controls in that AN patients were characterized by an attentional bias toward images of thin and fat bodies relative to images of social interactions. By contrast, another study [12] found both adolescent women with AN and bulimia nervosa (BN) as well as healthy control participants to preferentially process body parts often indexed as unattractive relative to other body parts.

When processing body parts pertaining to the self, one study [13] found adult women with AN to display a shorter gaze duration towards negatively valenced body parts, while in a recent study [14] adult AN and BN participants were characterized by longer and more frequent gazes towards negatively valenced body parts compared to control participants. Hence, the prior study points to an avoidance of disliked body parts, while the latter study suggests hypervigilance towards them. Notably, the inconsistencies found seem to be unrelated to the time course of the attentional processing of body-related cues. In fact, an eye-tracking study [15] which methodologically controlled for time course found no significant differences between early and later body-related attentional processing stages in eating disordered participants (AN, BN) and healthy controls. Nevertheless, this study found increased processing of the self-body compared to a weight-matched control body in adult AN patients, while control participants displayed a balanced pattern.

A somewhat neglected factor in research on body-related attentional processing is the effect of current mood. While several experimental studies have demonstrated that negative mood increases body size perception [16–18], there have been no experimental studies of the effects of current mood on attention allocation towards negatively and positively valenced body parts. One recent study, finding that selective attention towards negatively valenced body parts is weaker in AN patients with low compared to a high level of depression severity [14], suggests that the differential findings of previous studies might be attributable to participants’ current mood state.

The aim of the present study was to experimentally test the effects of mood on attention allocation towards self-defined most ugly and self-defined most beautiful body parts in female adolescent patients with AN compared to healthy controls (for readability, the terms *self-defined most ugly* and *self-defined most beautiful* body part will be abbreviated by *most ugly* and *most beautiful*). To this end, participants were given a positive and a negative mood induction at a one-week interval. After each mood induction, their eye movements were recorded during a body exposure in front of a mirror. Just as longitudinal research has demonstrated significant increases in body dissatisfaction in females compared to males in early adolescence [19, 20], adolescent females both with and without AN have been shown to be characterized by an increased bias towards disliked body parts [12]. Therefore, we expected that after the positive mood induction both adolescent groups would display longer and more frequent gazes towards their most ugly relative to their most beautiful body part. By contrast, after the negative mood induction, females with AN were expected to gaze significantly longer and more often at their most ugly body part relative to healthy control participants. Furthermore, we expected that compared to female healthy controls, AN patients would self-report a stronger decrease in appearance state self-esteem and a stronger increase in body dissatisfaction from pre to post mirror exposure following negative mood, but that no such effects would be found following positive mood.

## Materials and Methods

### Participants

Twelve adolescent female patients with AN and 12 adolescent female participants without a lifetime eating disorder (control group; CG) participated in the present study. The study was approved by the ethics committee of the Freiburg University Clinic (EUB 2009–083). Written informed consent was obtained from all parents; participants provided written assent.

Patients with AN were required to meet DSM-IV [21] criteria for AN, which was ascertained with the German version of the Structured Clinical Interview (SCID) for DSM-IV [22, 23]. Exclusion criteria for both the control group (CG) and the AN group included comorbid psychotic symptoms, current acute suicidal tendency, objections of the therapist responsible, considerable hypermetropia, and an age below 12 or above 18 years. An additional criterion for the CG was the presence of a current or lifetime eating disorder.

In total, 14 AN patients were interested in participating. Of those, two (14.3%) had to be excluded as they completed the diagnostic assessment session but did not show up to the scheduled eye tracking experiments. Thus, the final sample consisted of 12 AN patients, of whom 10 were in inpatient treatment and two in the department’s outpatient day clinic. According to DSM-IV [21], five AN patients (41.7%) were further classified as restricting type, five as binge-eating/purging type (41.7%) and two as Eating Disorder Not Otherwise Specified (EDNOS, AN-subtype; 16.6%). There were no significant group differences between AN patients restricting type and AN patients of the binge-purge type on age, BMI, overall psychopathology and body ratings (all *t*s [8] < 2.08, all *p*s > .07).

The CG was recruited via announcements at the local university clinic (i.e., among children of parents working at the clinic) and among acquaintances, matched for age and gender. In total, 15 females were invited and assessed with the SCID. Three (20.0%) had to be excluded: the first due to an occurring crisis after study inclusion (but prior to the eye tracking assessment), the second due to technical problems with the eye tracking device, and the third did not show up to the scheduled eye tracking experiments. The final control sample comprised 12 age and gender matched females.

### Questionnaires and Interviews

In addition to the initial diagnostic measure (SCID), the following self-report instruments were used: (1) The Youth Self-Report (YSR [24, 25]) is a psychometrically sound 112-item measure assessing the behavioral problems and competencies of children aged 11 to 18. It comprises eight syndromes designated as anxious/depressed, withdrawn/depressed, somatic complaints, social problems, thought problems, attention problems, rule-breaking behavior, and aggressive behavior. Higher scores reflect a higher level of psychopathology in the respective domain. Beyond that, three major scores of internalizing difficulties, externalizing difficulties and total difficulties can be calculated. In the present study, the internal consistency of the total difficulty score was very good with Cronbach’s α ranging from 0.90 (AN) to 0.94 (CG). (2) The Body Shape Questionnaire (BSQ [26, 27]) is a reliable and valid 34-item measure assessing body satisfaction. Higher scores represent higher body dissatisfaction. Here, internal consistency was high with Cronbach’s α of 0.93 in both groups. (3) In order to control for severity of depression, the German version of the Beck Depression Inventory-II (BDI-II [28, 29]) was administered. Internal consistency was high with Cronbach’s *α*_s_ of 0.93 and 0.85 for the AN group and the CG respectively. (4) Perceived beauty of body parts was assessed using a body part rating scale, developed for the present study. Participants were asked to rate the beauty of their shoulders, belly, décolleté, thighs, arms, hip, waist, lower legs and bosom on a 6-point rating scale (1 = ugly, 6 = beautiful). Thereafter they were asked to state their subjectively-felt most beautiful and most ugly body part. (5) The State Self-Esteem Scale (SSES [30, 31]) is a 20 item self-report instrument with good psychometric properties and sensitive to momentary changes in self-esteem that result from laboratory manipulations. It comprises three scales (performance, social and appearance self-esteem). Higher scores reflect higher momentary self-esteem in the respective domain. To reduce participants’ response burden, only the appearance subscale was administered. Participants completed the SSES four times over the course of the experiment (see procedure). Cronbach’s *α*_s_ for the appearance self-esteem subscale ranged from 0.73 to 0.93 for the AN group, and from 0.46 to 0.79 for the CG. (6) The Figure Rating Scale (FRS [32]) is a self-report measure used to assess body image disturbances. It consists of a series of nine schematic figures of increasing size with accompanying alphabetical ratings (A to I; numerically coded from 1 [smallest figure] to 9 [largest figure]). Participants were instructed to select a figure based on the following instruction: “Choose the figure that reflects how you feel at the moment”. Higher scores reflect a higher degree of body dissatisfaction. For the present study, the figures of the original FRS were substituted by the more modern and contemporary figures of the Contour Drawing Rating Scale [33]. It was administered seven times over the course of the experiment (see procedure). (7) In order to assess changes in mood, participants were given a 0–100 mm Visual Analogue Scale (VAS) asking “How do you feel now”, ranging from “depressed” to “happy”. It was administered seven times over the course of the experiment (see procedure).

### Mood Induction and Manipulation Check

To control for sequence effects, a cross-over design was chosen. All participants received both a positive and a negative mood induction within one week, with the order of the first and second induction counter-balanced across participants.

Positive and negative mood was induced by idiosyncratic autobiographical memories, an induction method which has previously been successful in the context of eating disorders (e.g., [34]). Participants were instructed to recall either a positive or a negative event, which had occurred in the weeks prior to the eye tracking assessments. A laboratory assistant prompted participants to mentally relive the event in their imagination. In addition, participants were asked a set of questions to instigate self-immersion in the imagined event (e.g., When did it happen? What exactly happened? Who else was present? What details do you recall, e.g., noises, odors …? How did it make you feel?), and instructed to make some notes on paper. The induction took about 15 to 20 min.

The manipulation check consisted of the mood VAS (see Questionnaires and Interviews), and by the following items anchored at 1 [worked easily] and 4 [did not work at all]: (1) *Did you succeed at imagining a vivid picture of the recalled event*? (2) *Did you succeed in re-living the associated emotions with the event*? (3) *Were you able to recall details of the surroundings*, *e*.*g*. *odors*, *noises and features*, *and did you imagine them vividly*? Lower scores indicated better task implementation. Internal consistency ranged between 0.62 and 0.63 for the whole sample, thus allowing aggregation of item scores.

### Procedure

The procedure of the present study was identical for patients and comparison participants. The diagnostic assessment (SCID) was conducted by a trained psychologist or psychiatrist at the inpatient center/outpatient day clinic. Diagnostic sessions were supervised by the clinic staff and one of the co-authors (CF). Firstly, parents of potential participants were contacted and given a detailed study description. During a second appointment, participants were informed about the study and parents gave written informed consent, participants provided written assent. Participants then filled out the questionnaires (YRS, FFB, BDI-II, Body Part Rating Scale) and height and weight were measured. After that, two eye tracking sessions were scheduled to take place within one week.

The two eye tracking sessions followed the same protocol: Participants were reassured that they could withdraw at any time without any disadvantage. After familiarization with the experimental setting, participants’ current mood (mood VAS), appearance state self-esteem (SSES) and body dissatisfaction (FRS) was assessed (time 1). Following this, they were informed about the upcoming mood induction and filled out the mood VAS and FRS once more (time 2). The laboratory assistant then started the first mood induction. At the end of the induction, participants again filled out the mood VAS, SSES and FRS (time 3), and completed the manipulation check questionnaire. Participants then changed into standardised underwear (cream panty and cream top) and were asked to remove their make up, after which they completed the mood VAS and FRS once more (time 4). They were then instructed to stand in front of a closed mirror (distance approx. 1.5 meters) behind a shoulder-high folding screen, while the experimenter mounted and calibrated the head-mounted eye-tracking device. The mirror was opened for participants to adapt to the sight of their head and the eye-tracking device in the mirror. Because of the screen, they could not yet see their bodies. When calibration was completed, the experimenter went out of sight, the eye tracking recording device was started and the screen removed. Participants were then instructed to look at the mirror. After three minutes, the eye-tracking device was removed and participants filled out the mood VAS, SSES and FRS once more (time 5). Having dressed, they completed the mood VAS and FRS again (time 6). Then participants were debriefed. Participants’ debriefing consisted of an exploration of their current mood in order to ensure their stability after participation in the experiment. Finally, they completed the mood VAS, SSES and FRS one last time (time 7).

### Data preprocessing

The eye-tracking recording consisted of 24 pictures per second and was analyzed using *Video Analyzer* (Senso Motoric Instruments [SMI]). For each picture, a laboratory assistant (blind to group membership and mood condition) identified the body part on which the cursor was set (representing the fixation direction of the eye). Individually, duration in sec (i.e. sum of the pictures coded for this body part divided by 24) and frequency (i.e. number of sequences of consecutive pictures coded for the same body part divided by the number of all such sequences per person) for each body part were computed. For each subject only the two body parts identified as most beautiful and most ugly were considered for the analyses.

### Statistical Analyses

The assumption of normality was satisfied in both groups for all descriptive scales (Kolmogoroff-Smirnoff *p*s > .08), mood VAS ratings (Kolmogoroff-Smirnoff p*s* > .20), nearly all SSES ratings (Kolmogoroff-Smirnoff *p*s > .06), except SSES time 3 and time 5 in the CG (Kolmogoroff-Smirnoff *p*s < .02), and most of the duration and frequency measures (Kolmogoroff-Smirnoff *p*s > .07). For FRS, the normality assumption was not met for more than half of the measurement points (Kolmogoroff-Smirnoff *p*s < .09) and was mostly not satisfied for body ratings (Kolmogoroff-Smirnoff *p*s < .05); therefore, nonparametric analyses were performed here.

Analyses of variance (ANOVA) were computed to analyze group differences on most descriptive variables. The Mann-Whitney U-Test was used to compare groups on body part ratings. The manipulation check item scores were analyzed by a 2 (Group: AN vs. CG) × 2 (Mood Induction: positive vs. negative) repeated measures ANOVA. Mood VAS before (time 2) and after induction (time 3) and before mirror exposure (time 4) was analyzed by a 2 (Group: AN vs. CG) × 2 (Mood Induction: positive vs. negative) × 3 (Time: time 2 vs. time 3 vs. time 4) repeated measures ANOVA followed by post-hoc pairwise comparisons in order to check the effect of mood induction. The course of mood VAS and FRS was analyzed by a 2 (Group: AN vs. CG) × 2 (Mood Induction: positive vs. negative) x 7 (Time: time 1 to time 7) repeated measures ANOVA, followed by pairwise contrasts of time (esp. time 1 vs. time 3; time 3 vs. time 4; time 4 vs. time 5) if feasible or if based on hypotheses. In order to account for non-normality, nonparametric pairwise comparisons were undertaken for FRS. The course of SSES was analyzed by a 2 (Group: AN vs. CG) × 2 (Mood Induction: positive vs. negative) × 4 (time: t1 vs. t3 vs. t5 vs. t7) repeated measures ANOVA.

For the eye movement data, separate analyses were undertaken for duration and frequency data with beauty ratings. Several 2 (Group: AN vs. CG) × 2 (Mood Induction: positive vs. negative) × 2 (Body Part: ugly/beautiful) repeated measures ANOVAs were conducted. In response to significant effects, pairwise comparisons were undertaken if feasible or if based on hypotheses.

## Results

### Sociodemographics and overall psychopathology

The groups did not differ in age (*F* [1, 22] < .001, *p* = .99). Expected differences however emerged on the BMI and body dissatisfaction scales (Table 1). Regarding general psychopathology, the groups did not differ on the YSR total score [24, 25], but patients with AN indicated more social withdrawal (*F* [1, 22] = 17.04, *p* < .001, *d* = 1.66) and higher anxious/depressed mood (*F* [1, 22] = 15.65, *p* < .001, *d* = 1.6) than participants in the CG. Accordingly, expected differences emerged on severity of depression, as assessed by the BDI-2 total score [28, 29].

**Table 1:** Sociodemographics and psychopathology of patients with anorexia nervosa (AN) and the healthy control group (CG).

Variable	AN (*n* = 12)	CG (*n* = 12)	Test statistics	*p*	d^1^
Gender	All female	All female			
Mean Age (years)	15.14 (1.55)	15.15 (1.57)	F (1, 22) < 0.001	0.99	< .01
BMI^2^	18.13 (1.46)	20.56 (2.29)	F (1, 22) = 9.66	0.005	1.27
BSQ total score	121.75 (30.76)	61.17 (18.02)	F (1, 22) = 34.66	0.001	2.4
Total Body Rating beautiful/ugly^3^	2.5 (1.17)	4.17 (0.72)	U = 126.00	0.001	
YSR total mean	51.17 (18.25)	40.00 (18.36)	F (1, 22) = 2.23	0.15	0.61
BDI-II total score	22.33 (12.09)	5.33 (4.44)	F (1, 22) = 20.9	0.001	1.87

Values in parentheses are standard deviations. BMI = body mass index (weight/height^2^); BSQ = Body Shape Questionnaire; YSR = Youth Self Report; BDI—II = Beck Depression Inventory—II.

^1^ As based on ranks, no effect size is reported for non-parametric tests;

^2^ BMI range/percentile [P] in the combined AN and AN-subtype group: BMI range = 14.7–20.3; P-range = P3 –P50. Note that only one patient in this group was at P50, all others ≤ P25. BMI range/percentile [P] in the CG: BMI range = 17.2–25/P50 –P90.

^3^ Single item, scored 1 = ugly to 6 = beautiful.

### Beauty ratings of body parts

With the exception of upper body parts, patients with AN rated their body parts as less beautiful than the CG (Table 2).

**Table 2:** Body part ratings.

Variable	AN (n = 12)	CG (n = 12)	test statistic (U, p)	*p*^1^
beauty ratings	M (SD)	M (SD)		
shoulders	3.67 (1.16)	4.42 (1.00)	U = 98.5	0.13
décolleté	3.17 (1.34)	4.33 (0.49)	U = 112	0.02
bosom	2.92 (1.51)	4.00 (0.74)	U = 110	0.03
arms^2^	3.25 (0.97)	4.83 (0.58)	U = 135	< 0.001
belly^2^	1.42 (0.67)	4.00 (1.21)	U = 139.5	< 0.001
waist^2^	2.75 (1.22)	4.33 (0.89)	U = 122	0.003
hip^2^	2.17 (0.72)	4.00 (0.60)	U = 140	< 0.001
thighs^2^	1.83 (1.03)	3.5 (1.24)	U = 121	0.004
lower legs^2^	2.83 (1.4)	4.42 (1.08)	U = 116	0.01

Notes. Due to multiple comparisons, statistical significance was set to *p* = .01.

^1^ As based on ranks, no effect size is reported for non-parametric tests;

^2^ = indicate significant group differences in body part rating.

Patients with AN and the CG did not differ in the body part indicated most beautiful (*χ*^*2*^ [8] = 15.29, *p* = .06) and most ugly (*χ*^*2*^ [5] = 7.78, *p* = .17) (Table 3). In both groups, the body parts selected as most ugly were mainly the belly and thighs (chosen as most ugly by 100% of AN and 58.3% of CG). In the selection of most beautiful body parts, more variation occurred, though mainly body parts located in the upper part of the body, i.e. shoulders, décolleté, bosom, arms or belly, were chosen. Ratings of the most ugly body part were lower in patients with AN than CG, indicating higher body dissatisfaction (Table 3).

**Table 3:** Selected body parts and individual ratings.

Variable	AN(n = 12)	CG(n = 12)	test statistics	*p*^1^
Most beautiful body part (*N*, *%*)				
shoulders	3 (25)	1 (8.3)	χ² = 15.29, df = 8	0.06
décolleté	5 (41.7)	2 (16.7)		
bosom	0	1 (8.3)		
arms	0	3 (25)		
belly	0	2 (16.7)		
waist	3 (25)	0		
hip	0	2 (16.7)		
thighs	0	1 (8.3)		
lower legs	1 (8.3)	0		
ratings^2^ (M, SD)	3.92 (1.24)	4.83 (.58)	U = 104.5	0.06
Most ugly body part (*N*, *%*)				
shoulders	0	1 (8.3)	χ² = 7.78, df = 5	0.17
décolleté	0	1 (8.3)		
bosom	0	2 (16.7)		
arms	0	0		
belly	7 (58.3)	2 (16.7)		
waist	0	0		
hip	0	0		
thighs	5 (41.7)	5 (41.7)		
lower legs	0	1 (8.3)		
ratings^2^ (M, SD)	1.42 (.79)	2.83 (.94)	U = 124	0.002

Notes. Due to multiple comparisons, statistical significance was set to p = .01.

^1^ As based on ranks, no effect size is reported for non-parametric tests;

^2^ = Individuals' ratings on a 6-point rating scale (1 = ugly, 6 = beautiful).

### Mood: manipulation check and course

The repeated measures ANOVA on the MC item scores revealed no significant main effect of Group (*F* [1, 22] = .54, *p* = .47, η² _part_ = .03), but a significant main effect of Mood (*F* [1, 22] = 8.96, *p* = .007, η² _part_ = .29). MC item scores were significantly lower in the positive (*M* = 1.72, *SD* = 0.51) than in the negative mood (*M* = 2.10, *SD* = 0.52) induction, thus indicating a better task implementation in the positive mood induction condition. There was no significant interaction of Group × Mood (*F* [1, 22] = .60, *p* = .45, η² _part_ = .03).

**Fig 1:**
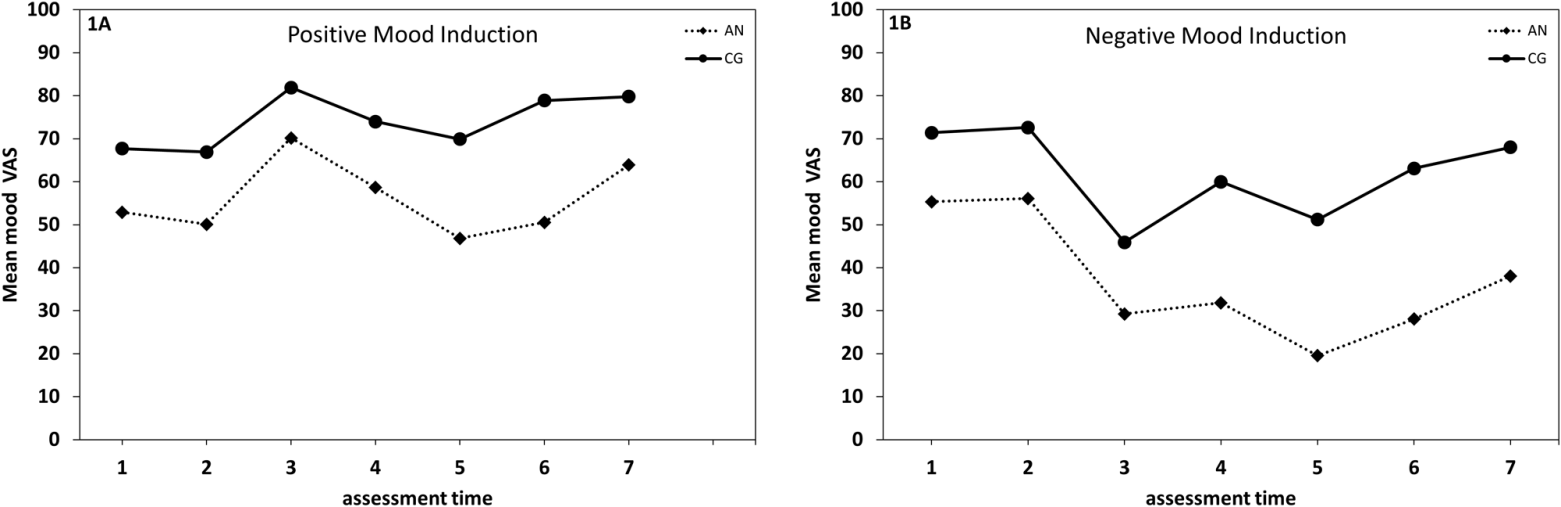
Time course of mood. Time course of mood in the positive mood induction condition (Fig 1A) and the negative mood induction condition (Fig 1B) in females with anorexia nervosa (AN) and females in the healthy control group (CG). Time points: 1 = at baseline, 2 = prior to the mood induction, 3 = after the mood induction, 4 = after changing, 5 = after mirror exposure, 6 = having re-dressed, 7 = at termination. Scale: VAS = Visual Analogue Scale (0–100 mm), ranging from “depressed” to “happy”.

There were no significant group differences with regard to the self-reported mood VAS at time 1 (positive mood induction: *t* [19] = -1.69, *p* = .11, *d* = -.74; negative mood induction: *t* [19] = -1.81, *p* = .09, *d* = .57). In order to check the effect of the mood induction, mood VAS prior to the mood induction (time 2), after the induction (time 3) and prior to the mirror exposure (time 4) were compared. This resulted in a main effect of Mood Induction (*F* [1, 22] = 18.71, *p* < .001, η² _part_ = .46), a main effect of Time (*F* [2, 44] = 5.39, *p* = .008, η² _part_ = .20), a main effect of Group (*F* [1, 22] = 13.39, *p* = .001, η² _part_ = .38), as well as the expected interaction of Induction × Time (*F* [2, 44] = 63.37, *p* < .001, η² _part_ = .74; Fig 1A and 1B). Notably, there were no significant mood VAS differences immediately prior to the mood induction (time 2; *t* [23] = -1.24, *p* = .23, *d* = .25).

To disentangle the interaction of Induction × Time, we conducted paired t-tests separately for each induction condition. In the negative mood induction condition, there was a significant mood VAS decrease from prior (time 2) to after (time 3) the negative mood induction (*t* [23] = 9.35, *p* < .001, *d* = -1.92). There was, however, a significant mood increase from after the negative mood induction (time 3) to the time after which participants had changed their clothes (i.e., immediately prior to the mirror exposure/time 4; *t* [23] = -3.45, *p* = .002, *d* = .72), though mood remained more negative than before induction (time 2; *t* [23] = 5.41, *p* < .001, *d* = -1.11). In the positive mood induction condition, there was a significant mood increase from prior to (time 2) to after the positive mood induction (time 3; *t* [23] = -7.48, *p* < .001, *d* = 1.51), but a significant decrease from after the positive mood induction (time 3) to when participants had changed their clothes (i.e., immediately prior to the mirror exposure/time 4; *t* [23] = 3.96, *p* = .001, *d* = -.81). Thus, the effect of the positive mood induction was no longer significant prior to the mirror exposure (*t* [23] = -1.98, *p* = .06, *d* = .41). Based on this instability of the positive mood induction, individual mood VAS scores were inspected, identifying three cases (one AN, two CG), in which mood induction apparently did not work, with VAS ratings differing by about 20 points and opposite to the expected direction between baseline (time 1) and prior to mirror exposure (time 4). After omitting these cases, the mood induction remained significant between mood induction (time 2) and the time point prior to the mirror exposure (time 4) in both induction conditions (*t*s [20] ≥ 2.92, *p*s ≤ .008, *d*s ≥ .66). The omitted cases did not differ from valid cases in age (*t* [22] = .87, *p* = .40), BMI (*t* [22] = 1.10, *p* = .29) or on any questionnaire scores (*t*s [22] ≤ .61, *p*s ≥ .55). The following analyses also omit these discrepant cases.

The course of mood VAS was comparable in both groups (Fig 1A and 1B). The 2 (Group: AN, CG) × 2 (Mood Induction: positive, negative) × 7 (Time: time 1 to time 7) repeated measures ANOVA yielded significant main effects of Group (*F* [1, 19] = 16.06, *p* = .001, η² _part_ = .46), Mood Induction (*F* [1, 19] = 14.00, *p* = .001, η² _part_ = .42) and Time (*F* [6, 114] = 12.14, *p* < .001, η² _part_ = .39). There were further significant interactions of Group × Time (*F* [6, 114] = 4.31, *p* = .001, η² _part_ = .19), and of Induction × Time (*F* [6, 144] = 22.47, *p* < .001, η² _part_ = .54). The mood induction was successful, as the mood VAS at time 3 and all following time points was higher in the positive induction than in the negative induction condition (*t*s [20] ≥ 3.83, *p*s ≤ .001, *d*s ≥ .84), whereas no differences occurred at time 1 or time 2 (*t*s [20] ≤ -1.15, *p*s ≥ .27, *d*s ≤ .25).

In the positive induction condition, mood significantly increased from prior (time 2) to after the induction (time 3; *t* [20] = -8.21, *p* < .001, *d* = 1.81), significantly decreased thereafter (i.e., from time 3 to time 4; *t* [20] = 3.1, *p* = .006, *d* = -.68) and did not change after the mirror exposure (i.e., from time 4 to time 5; *t* [20] = -1.96, *p* = .06, *d* = -.43). Further, there was no mood change from after the mirror exposure (time 5) to after re-dressing (time 6; *t* [20] = -1.97, *p* = .07, *d* = -.74), but there was a significant increase in mood thereafter, by experiment termination (i.e., time 7; *t* [20] = -3.71, *p* = .001, *d* = .81).

Greater mood variability was found in the negative mood induction condition. There was a significant decrease in mood VAS scores from prior to (time 2) to after the induction (time 3; *t* [20] = 8.28, *p* < .001, *d* = -1.81), a significant increase thereafter (i.e., from time 3 to time 4; *t* [20] = -3.2, *p* = .005, *d* = .71), and a significant decrease from prior (time 4) to after the mirror exposure (time 5; *t* [20] = 3.97, *p* = .001, *d* = -.87). Mood VAS scores significantly increased from after the mirror exposure (time 5) to after re-dressing (time 6; *t* [20] ≥ -3.69, *p* ≤ .001, *d* ≥ .82), and a further significant increase occurred from after re-dressing (time 6) to experiment termination (time 7; *t* [20] ≥ -5.99, *p* ≤ .001, *d* ≥ .88).

In the positive mood induction condition the CG reported higher mood VAS than the AN group only prior to the induction (time 2: *t* [19] = 2.41, *p* = .03, *d* = 1.05) and after the mirror exposure (time 5 and time 6: *t*s [19] ≥ 3.08, *p*s ≤ .006, *d*s ≥ 1.35). Under the negative mood induction, group differences were more pronounced with participants in the CG reporting higher mood VAS than AN patients at all time points (*t*s(19) ≥ 2.17, *p*s ≤ .05, *d*s ≥ .92), except at time 1. This indicates higher overall mood impairment in the AN group.

### Self-reported body image measures

**Fig 2:**
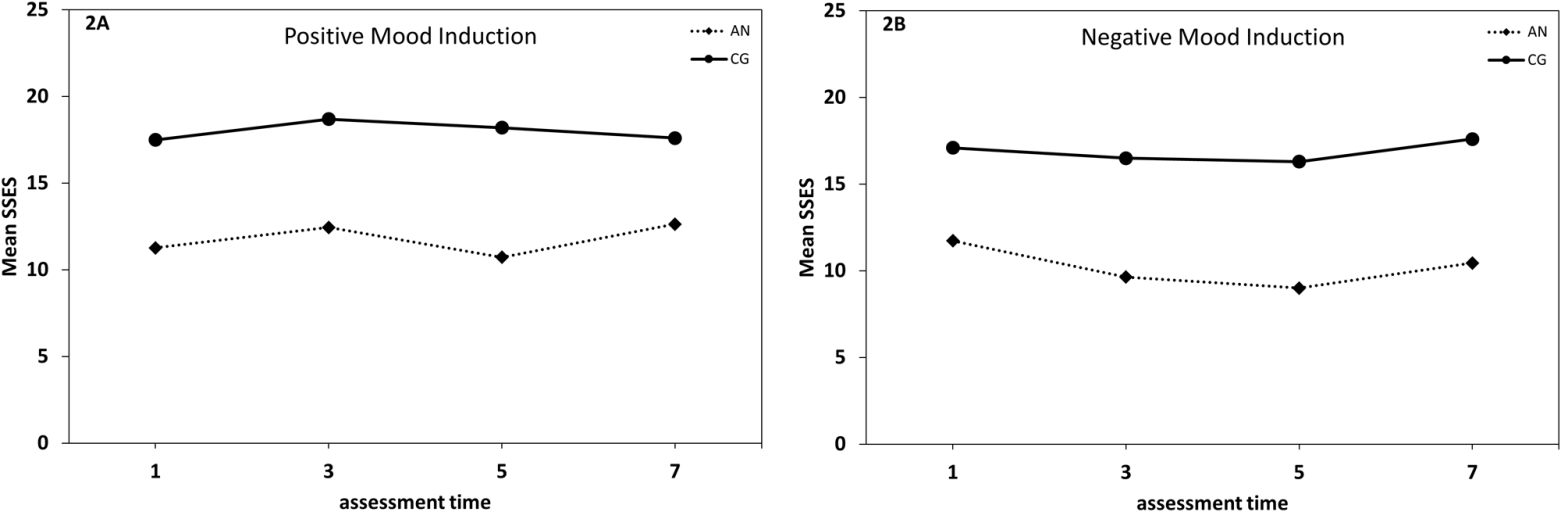
Time course of appearance state self-esteem. Time course of mood in the positive mood induction condition (Fig 2A) and the negative mood induction condition (Fig 2B) in females with anorexia nervosa (AN) and a healthy control group (CG). Time points: 1 = at baseline, 3 = after the mood induction, 5 = after mirror exposure, 7 = at termination. SSES = State Self-Esteem Scale, appearance subscale.

The 2 (Group: AN, CG) × 2 (Mood Induction: positive, negative) × 4 (Time: 1 vs. t3 vs. t5 vs. t7) repeated measures ANOVA for the appearance subscale of the SSES resulted in significant main effects of Group (*F* [1, 19] = 32.64, *p* < .001, η² _part_ = .63), Mood Induction (*F* [1, 19] = 11.36, *p* = .003, η² _part_ = .37), and Time (*F* [3, 57] = 5.72, *p* = .002, η² _part_ = .23; Fig 2A and 2B). There was also a significant interaction of Induction × Time (*F* [3, 57] = 8.43, *p* < .001, η² _part_ = .31), while the expected threefold interaction of Group × Induction × Time did not reach significance (*F* [3, 57] = 2.03, *p* = .12, η² _part_ = .10). As expected, participants in the CG reported higher appearance self-esteem scores than patients with AN throughout the experiment (*t*s ≥ 4.01, *p*s ≤ .001, *d*s ≥ 1.75). From time 1 to time 3, there was a significant overall decrease in appearance self-esteem in the negative mood induction condition (*t* [20] = 3.36, *p* = .003, *d* = -.73) and a significant increase after the positive mood induction (*t* [20] = -3.99, *p* = .001, *d* = .89). This overall difference in appearance SSES between the two mood induction conditions remained significant until the end of the experiment (time 7; *t*s [20] ≥ 3.58, *p*s ≤ .002, *d*s ≥ .77).

In the positive induction condition, there was a significant decrease from pre to post mirror exposure (time 3 vs. time 5) in appearance self-esteem (*t* [20] = 2.41, *p* = .03, *d* = -.51), but no change in SSES scores in the negative mood induction condition (*t* [20] = 1.25, *p* = .23, *d* = -.26). In both mood induction conditions there was an increase in appearance self-esteem from after the mirror exposure (time 5) to experiment termination (time 7; *t*s [20] ≥ -2.45, *p*s ≤ .02, *d*s ≥ .54).

**Fig 3:**
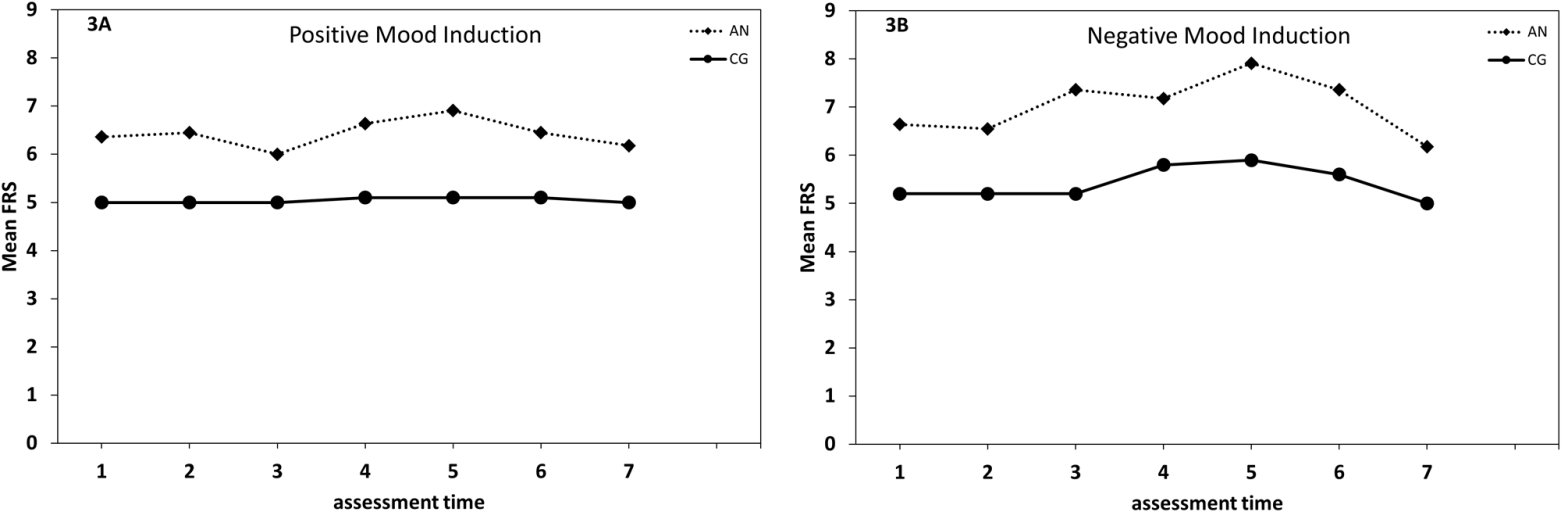
Time course of the Figure Rating Scale. Time course of mood in the positive mood induction condition (Fig 3A) and the negative mood induction condition (Fig 3B) in females with anorexia nervosa (AN) and a healthy control group (CG). Time points: 1 = at baseline, 2 = prior to the mood induction, 3 = after the mood induction, 4 = after changing, 5 = after mirror exposure, 6 = having re-dressed, 7 = at termination. FRS = Figure Rating Scale.

For the FRS (Fig 3A and 3B), the 2 (Group: AN, CG) × 2 (Mood Induction: positive, negative) × 7 (Time: time 1 to time 7) repeated measures ANOVA resulted in significant main effects of Group (*F* [1, 19] = 9.04, *p* = .007, η² _part_ = .32), Mood Induction (*F* [1, 19] = 14.25, *p* = .001, η² _part_ = .43), and Time (*F* [1, 114] = 9.91, *p* < .001, η² _part_ = .34), as well as a significant interaction of Mood Induction × Time (*F* [6, 114] = 2.88, *p* = .01, η² _part_ = .13). The expected threefold interaction of Group × Mood Induction × Time just failed to reach significance (*F* [6,114] = 1.84, *p* = .10, η² _part_ = .09).

Specifically, patients with AN generally choose bigger silhouettes than participants in the CG, except after the positive mood induction (time 3; Wilcoxon = 34.5, *p* = .15). While the chosen silhouettes in the CG remained quite stable in both mood induction conditions (all *p*s ≥ .13), patients with AN showed more variability. In the positive mood induction condition patients with AN showed a trend to choose thinner silhouettes from prior to (time 2) to after the mood induction (time 3; Wilcoxon = 0, *p* = .06), followed by an increase in chosen silhouette size from after the mood induction (time 3) to after changing clothing (time 4; Wilcoxon = 21, *p* = .02). All other within-comparisons in the positive mood induction condition were not significant (all *p*s ≥ .26). In the negative mood induction condition, patients with AN chose bigger silhouettes after the negative mood induction (i.e., time 2 to time 3; Wilcoxon = 28, p = .01) and after the mirror exposure (i.e., time 4 to time 5; Wilcoxon = 40, *p* = .02). All other within-comparisons in the negative mood induction condition were not significant (all *p*s ≥ .26).

### Gaze duration

**Fig 4:**
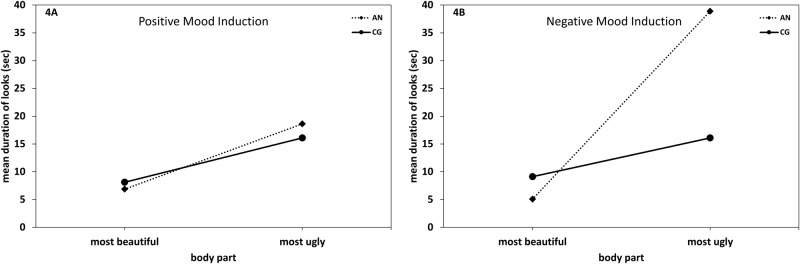
Time spent looking at the most beautiful and most ugly body part in the positive and negative mood conditions. Eye movement pattern in patients with anorexia nervosa (AN) and in the healthy control group (CG). Duration (in seconds [sec]) of looking at the most beautiful/ugly body part in the positive [4A] and the negative [4B] mood induction condition.

The 2 (Group: AN, CG) × 2 (Mood Induction: positive, negative) × 2 (Body Part: ugly vs. beautiful) repeated measures ANOVA resulted in a significant main effect of Body Part (*F* [1, 19] = 33.96, *p* < .001, η² _part_ = .64), as well as significant interactions of Group × Mood Induction (*F* [1, 19] = 5.53, *p* = .03, η² _part_ = .23) and Group × Body Part (*F* [1, 19] = 13.05, *p* = .002, η² _part_ = .41). The expected threefold interaction of Group × Mood Induction × Body Part just failed to reach significance (*F* [1, 19] = 3.65, *p* = .07, η² _part_ = .16). Hypotheses-based post-hoc tests (Fig 4A and 4B) showed that patients with AN looked longer at the most ugly than the most beautiful body part both in the positive and the negative mood induction conditions (*t*s [10] ≥ -2.93, *p*s ≤ .02, *d*s ≥ 1.65), while participants in the CG looked longer at the most ugly than the most beautiful body part in the positive mood induction condition (*t* [9] = - 2.61, *p* = .03, *d* ≥ 1.52), but not in the negative mood induction condition (*t* [9] = -.99, *p*s ≤ .35, *d*s ≥ .83). Regarding between-group differences, patients with AN looked longer at their most ugly body part than participants in the CG in the negative mood induction condition (*t* [19] = 2.6, *p* = .02, *d* = 1.14). There were no significant group differences with regard to beautiful body parts in either induction condition (*ts* [19] ≤ 1.37, *p* ≥ .19, *ds* ≤ -0.59), nor significant group differences with regard to the most ugly body part in the positive mood induction condition (*t* [19] = .68, *p* = .50, *d* = 0.30). Analogous results were obtained in non-parametric Mann-Whitney-U-Tests.

### Gaze frequency

**Fig 5:**
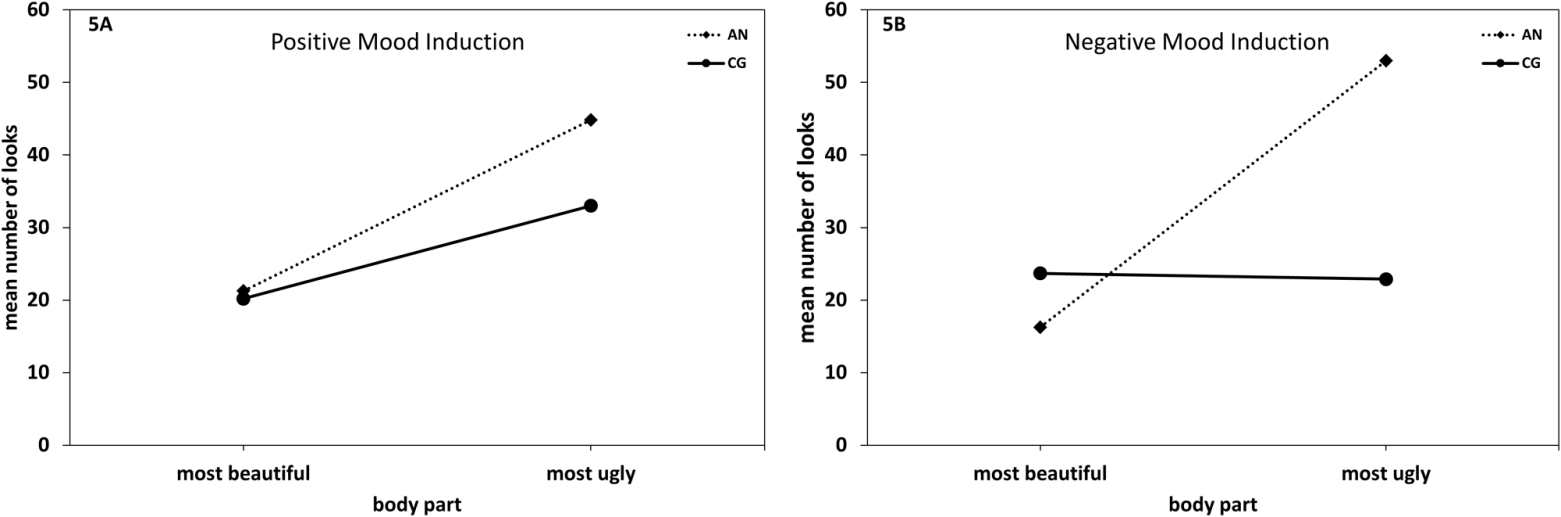
Number of looks towards the most beautiful and most ugly body part in the positive and negative mood conditions. Eye movement pattern in patients with anorexia nervosa (AN) and in the healthy control group (CG). Number of looks at most beautiful/ugly body part in the positive [5A] and the negative [5B] mood induction condition.

The 2 (Group: AN, CG) × 2 (Mood Induction: positive, negative) × 2 (Body Part: ugly vs. beautiful) repeated measures ANOVA resulted in a significant main effect of Body Part (*F* [1, 19] = 24.75, *p* < .001, η² _part_ = .57), a significant interaction of Group × Body Part (*F* [1, 19] = 11.04, *p* = .004, η² _part_ = .37) and the expected threefold interaction of Group × Mood Induction × Body Part (*F* [1, 19] = 6.04, *p* = .02, η² _part_ = .24). As can be seen in Fig 5A and 5B, patients with AN looked more often at their most ugly body part than their most beautiful body part in both mood induction conditions (*t*s [10] ≥ 3.61, *p*s ≤ .005, *d*s ≥ 1.09). By contrast, the CG showed a trend to significance in the positive mood induction condition (*t* [9] = -2.19, *p* = .06, *d* = - 1.37), but a balanced pattern in the negative mood induction condition (*t* [9] = 0.22, *p* = .80, *d* = .11). Furthermore, only in the negative mood induction condition did patients with AN gaze more frequently at their most ugly body part than participants in the CG (*t* [19] = 3.55, *p* = .002, *d* = 1.55). Analogous results were obtained in non-parametric Mann-Whitney-U-Tests.

### Correlations of gazes and self-reported body dissatisfaction

There were no significant correlations (spearman) across groups between gaze parameters and FRS scores in the positive mood induction condition (all *r*s ≤ -.33, all *p*s ≥ .14). Within groups, there was a significant correlation (spearman, one-tailed) in the positive mood induction condition between FRS scores assessed after the mirror exposure (time 5) and gaze frequency towards the most ugly body part in the AN group (*r* = -.63, *p* = .05). In the CG, gaze frequency towards the most ugly body part correlated (spearman; one-tailed) significantly with FRS scores assessed after the mood induction (time 3; *r* = -.56, *p* = .004) and FRS scores assessed after the mirror exposure (time 5; *r* = -.64, *p* = .002) in the positive mood induction condition. All other correlations in the positive mood induction condition were not significant (all *r*s ≤ .33; all *p*s > .10).

In the negative mood induction condition, there were significant correlations (spearman; one-tailed) between gaze duration and gaze frequency towards the most ugly body part and FRS scores assessed after the mood induction (time 3; duration: *r* = .64, *p* = .001/frequency: *r* = .63, *p* = .003) and FRS scores assessed after the mirror exposure (time 5; *r* = .58, *p* = .003; *r* = .55, *p* = .001). All other correlations in the negative mood induction condition were not significant (all *r*s ≤ .35, all *p*s > .10).

## Discussion

The aim of the present experiment was to test the causal effects of mood on attentional processing of the self-body in female adolescents with a diagnosis of AN in comparison to a healthy CG. Based on previous results [12, 14], it was hypothesized that after a positive mood induction females both with and without AN would be characterized by longer and more frequent gazes towards their most ugly relative to their most beautiful body part. By contrast, after a negative mood induction, patients with AN were expected to gaze significantly longer and more often towards the most ugly body part relative to controls. Furthermore, it was hypothesized that compared to the CG, females with AN would report a larger decrease in appearance state self-esteem and a larger increase in body dissatisfaction from pre to post mirror exposure following negative mood induction, but not following positive mood induction.

Our hypotheses were partially confirmed. Specifically, in the positive mood induction condition, AN participants looked significantly longer and more often at their most ugly than at their most beautiful body part. A similar gaze pattern was found for participants in the CG. In the negative mood induction condition, however, this pattern was found only in the AN group; though, gaze frequency and duration of females in the CG were evenly distributed between the most ugly and the most beautiful body part. In addition, in the negative mood induction condition gaze frequency and duration towards the most ugly body part was significantly higher in the AN group than in the CG, while there was no such group difference in the positive mood induction condition.

Puberty, and its associated physical changes, makes young teenagers highly critical of themselves. As such, previous research yields evidence of a decline in self-esteem around the ages of 13 to 15 [35] and in body satisfaction in early adolescent girls [19, 20]. The gaze pattern found in the positive mood induction in both adolescent groups may therefore reflect the overvaluation of shape and weight typical of this time period, in which kidlike/juvenile body parts mature to an adult body. In line with this, theoretical models [36] postulate that body-related cognitive biases are a function of disordered body schemata, rather than disordered eating. From a developmental perspective, prospective studies should investigate whether there are prior psychopathological patterns and trajectories of body-related attentional processing. This is especially important as previous comparisons of attentional processing of the self-body in adults yielded evidence of balanced attention distribution between positively and negatively valenced body parts in women without an eating disorder, and an attentional bias towards negatively valenced body parts in women with AN [14].

In the AN group attention towards the most ugly body part was stronger in the negative than in the positive mood induction condition. In the CG, the bias towards the most ugly body part was somewhat weaker in the negative than in the positive mood induction condition. Previous experimental studies have shown that individuals frequently and consciously retrieve positive autobiographical memories as a means of repairing negative mood following negative mood inductions [37–39]. In addition, experimental studies [40, 41] have found that such mood repair is not evident in dysphoric individuals and those characterized by current depression. Consequently, healthy participants’ reduced attention towards negatively valenced body parts in the negative (compared to the positive) mood induction condition may have constituted some form of mood repair (or counter-regulation), which was not evident in the AN group. In fact, in the negative mood induction condition there was a significant decrease in mood from experiment entry to experiment termination in the AN group, while mood was again restored in the CG. From a clinical perspective, the increased attentional bias for the most negatively valenced body parts during the experience of negative mood may lead to a persistence and aggravation of AN patients’ negative body image, as qualifying additional information or neutral and positive body information are not attended to and are thus neglected. This, in turn, may increase the frequency of periods of dysphoria, thereby maintaining and strengthening the negative body schema.

Contrary to our hypothesis, there was no differential course of appearance self-esteem between AN patients and the CG following the mood induction. Further, the mood induction affected appearance self-esteem comparably in both groups. Previous studies have shown that the stability of self-esteem is rather low in early adolescence [42]. We used the SSES to assess short-term changes in self-esteem and found that appearance self-esteem is highly affected by current mood and the exposure to one’s body. This converges with experimental studies showing a close linkage between low self-esteem and increased body dissatisfaction [43, 44]. In the present study, the CG self-reported higher appearance self-esteem relative to AN patients throughout. However, in both groups appearance self-esteem was highly affected by the mood induction. Future studies should therefore independently test the effects of mood and appearance self-esteem on the attentional processing of the self-body by selectively manipulating one variable (but not the other).

Comparable to the course of appearance self-esteem, there was no differential course of body dissatisfaction (FRS scores) between AN patients and the CG following the mood induction. Patients with AN generally chose larger silhouettes than participants in the CG. While after the negative mood induction and after the mirror exposure AN participants self-reported an increase in silhouette size, silhouette ratings were less affected by mood in the CG, possibly indicating a more stable body-related concept in this group.

Notably, across groups there were no significant correlations between gaze parameters and body dissatisfaction following the positive mood induction. On the one hand this could be due to the rather low ecological validity of the FRS [45], which may artificially reduce the variance necessary to assess body dissatisfaction [46]. Arguing against this, there were significant correlations across groups in the negative mood induction condition. Specifically, the longer and more often participants gazed towards the most ugly body part, the higher their self-reported body dissatisfaction both after the negative mood induction and the mirror exposure. In the positive mood induction condition there were further significant negative within-group correlations between gaze frequency towards the most ugly body part and FRS scores following the mirror exposure in both groups, and an additional significant and negative correlation between gaze frequency and FRS scores following the mirror exposure in the CG. From a theoretical perspective [9, 36], increased attentional bias toward the most negatively valenced body part may lead to the persistence and aggravation of negative body image, more so in the course of negative mood than in the course of positive mood.

Several limitations have to be mentioned. Most of all, the sample size was very small and only enabled the detection of group differences based on large effects [47]. Some overall ANOVAS just failed to reach significance. Even though hypotheses-based post-hoc tests yielded differential group and condition effects in conservatively conducted two-tailed tests, future studies are required to replicate these findings in order to draw meaningful clinical conclusions. Second, the lack of a neutral mood condition limits conclusions with regard to the influence of negative (and positive) mood on the attentional processing of the self-body. Therefore, future studies should compare gaze patterns in positive or negative mood with a neutral condition. Another limitation concerns the mood induction itself. Even though both groups experienced significant and lasting mood change following the mood induction in the expected direction, participants self-reported better implementation of the positive than the negative induction. Hence, future studies should ensure better comparability between the two mood induction conditions. Finally, some of the AN patients were tested in the course of their treatment and had therefore already started weight restoration. As such, it remains unclear whether different gaze patterns would emerge in a sample of AN patients not in the phase of weight restoration.

While the correlational nature of the study precludes drawing conclusions about the causal status of attentional biases for unattractive body parts in explaining body dissatisfaction, first experimental evidence [48] provides support for its role in the etiology and maintenance of body dissatisfaction. As such, training highly body-dissatisfied women to selectively attend to their three most self-defined attractive body parts has been shown to significantly increase self-reported body and weight satisfaction. The present findings emphasize that attentional biases could be a cognitive mechanism contributing to body dissatisfaction, especially during negative mood. From a clinical perspective, therefore, it will be important to determine whether attentional bias modification training also stabilizes attentional processing towards self-liked body parts during negative mood.
